# Supplementary material for: End Sequence Analysis Toolkit (ESAT) expands the extractable information from single-cell RNA-seq data
Source: Genome Res. 2016 Oct;26(10):1397–410. doi: 10.1101/gr.207902.116 (PMC5052061; doi:10.1101/gr.207902.116)
Supplement: Supplemental Material [file supp_gr.207902.116_Supp_Materials.tar.gz › ESAT-esat_v0.1/doc/html/classbroad_1_1pda_1_1seq_1_1graph_1_1_path-members.html]

ESAT: Member List


ESAT


- Main Page
- Related Pages
- Packages
- Classes
- Files

- Class List
- Class Index
- Class Hierarchy
- Class Members

All Classes Namespaces Files Functions Variables Enumerator Pages

- broad
- pda
- seq
- graph
- Path

broad.pda.seq.graph.Path Member List

This is the complete list of members for broad.pda.seq.graph.Path, including all inherited members.

|  |  |  |
| --- | --- | --- |
| addEdge(BubbleEdge edge) | broad.pda.seq.graph.Path |  |
| addEdges(Collection< BubbleEdge > edges) | broad.pda.seq.graph.Path |  |
| addPairedEnd(Alignment align) | broad.pda.seq.graph.Path |  |
| addPairedEndEdges(Collection< Alignment > pairedEndEdges2) | broad.pda.seq.graph.Path |  |
| compareTo(Path b) | broad.pda.seq.graph.Path |  |
| copy() | broad.pda.seq.graph.Path |  |
| equals(Object o) | broad.pda.seq.graph.Path |  |
| getAlignment() | broad.pda.seq.graph.Path |  |
| getChromosome() | broad.pda.seq.graph.Path |  |
| getCoverage() | broad.pda.seq.graph.Path |  |
| getEdgeList() | broad.pda.seq.graph.Path |  |
| getEdges() | broad.pda.seq.graph.Path |  |
| getEnd() | broad.pda.seq.graph.Path |  |
| getEndVertex() | broad.pda.seq.graph.Path |  |
| getGraph() | broad.pda.seq.graph.Path |  |
| getLocalLambda() | broad.pda.seq.graph.Path |  |
| getNodeCount(Annotation v1) | broad.pda.seq.graph.Path |  |
| getNumberOfEdges() | broad.pda.seq.graph.Path |  |
| getOrientation() | broad.pda.seq.graph.Path |  |
| getOverlappingNodes(Alignment read) | broad.pda.seq.graph.Path |  |
| getPairedEndEdges() | broad.pda.seq.graph.Path |  |
| getPercentConsistent(EmpiricalDistribution pairedInsertDistribution) | broad.pda.seq.graph.Path |  |
| getScore() | broad.pda.seq.graph.Path |  |
| getSize() | broad.pda.seq.graph.Path |  |
| getSortedNodes() | broad.pda.seq.graph.Path |  |
| getSpliceWeight() | broad.pda.seq.graph.Path |  |
| getStart() | broad.pda.seq.graph.Path |  |
| getStartVertex() | broad.pda.seq.graph.Path |  |
| getWeight() | broad.pda.seq.graph.Path |  |
| hashCode() | broad.pda.seq.graph.Path |  |
| isToComplex() | broad.pda.seq.graph.Path |  |
| overlaps(Path leftPath) | broad.pda.seq.graph.Path |  |
| overlapsLeft(Alignment read) | broad.pda.seq.graph.Path |  |
| overlapsRight(Alignment read) | broad.pda.seq.graph.Path |  |
| Path(Collection< BubbleEdge > edges, double spliceWeight) | broad.pda.seq.graph.Path |  |
| Path(Collection< BubbleEdge > edges, Transformer< BubbleEdge, Number > transformer, double spliceWeight) | broad.pda.seq.graph.Path |  |
| Path(double spliceWeight) | broad.pda.seq.graph.Path |  |
| Path() | broad.pda.seq.graph.Path |  |
| Path(Gene gene, ChromosomeWithBubblesJGraphT graph) | broad.pda.seq.graph.Path |  |
| removeEdge(BubbleEdge edge) | broad.pda.seq.graph.Path |  |
| setIsToComplex(boolean isToComplex) | broad.pda.seq.graph.Path |  |
| setLocalLambda(double localLambda) | broad.pda.seq.graph.Path |  |
| setSpliceWeight(double weight) | broad.pda.seq.graph.Path |  |
| toFilledInGene() | broad.pda.seq.graph.Path |  |
| toGene() | broad.pda.seq.graph.Path |  |
| toGene(double[] extraFields) | broad.pda.seq.graph.Path |  |
| toString() | broad.pda.seq.graph.Path |  |


---

Generated on Fri Aug 15 2014 15:11:02 for ESAT by  

 1.8.7
